# Predictive value of preoperative neutrophil-to-lymphocyte ratio for prognosis in aortic dissection patients without hypertension following TEVAR

**DOI:** 10.3389/fragi.2026.1875447

**Published:** 2026-07-09

**Authors:** Shun Yu, Xinghua Liu, Kunyu Guan, Liang Chen, Hao Zhang, Dengqun Sun, Yanjun Sun, Lixue Zhu, Lijun Weng, Biao Wu

**Affiliations:** 1 Department of Vascular Surgery, Changhai Hospital, Shanghai, China; 2 Department of Vascular Surgery, 920th Hospital of Joint Logistics Support Force, PLA, Kunming, Yunnan, China; 3 Pediatric, Changhai Hospital, Shanghai, China; 4 Department of General Surgery, The Chinese People’s Armed Police Forces Anhui Provincial Corps Hospital, Hefei, China; 5 Department of Medical Imaging, The Chinese People’s Armed Police Forces Anhui Provincial Corps Hospital, Hefei, China

**Keywords:** aortic dissection, neutrophil-to-lymphocyte ratio, NLR, TEVAR, thoracic endovascular aortic repair

## Abstract

**Background:**

The neutrophil-to-lymphocyte ratio (NLR) serves as a potential inflammatory biomarker for aortic dissection (AD), with elevated levels predicting adverse postoperative outcomes. Nevertheless, the prognostic significance of preoperative NLR in non-hypertensive AD patients receiving thoracic endovascular aortic repair (TEVAR) remains undefined.

**Objective:**

To evaluate the predictive value of preoperative NLR for clinical prognosis in this specific cohort following TEVAR.

**Methods:**

This study retrospectively analyzed 116 non-hypertensive AD patients undergoing TEVAR at our medical center (December 2014–December 2020). Receiver operating characteristic (ROC) curve analysis determined the optimal NLR cutoff, area under the curve (AUC), and 95% confidence interval (CI) for predicting the primary endpoint (all-cause mortality). Patients were stratified into low-NLR and high-NLR groups based on the cutoff. Survival differences were assessed using *Kaplan-Meier* analysis with *log-rank* testing. Associations between NLR and mortality risk were evaluated via univariate and multivariate Cox regression.

**Results:**

The cohort comprised 77.6% males, with 52.6% aged >55 years, 30.2% reporting smoking history, 13.8% alcohol use, and 14.7% comorbidities. Anatomical classification included Stanford type A (5.2%) and type B (94.8%); temporal classification was acute (12.1%), subacute (30.2%), and chronic (57.8%). Branch artery reconstruction was performed in 47.4% of patients. Postoperative outcomes included all-cause mortality (1.7%), dissection recurrence (3.4%), endoleak (6.0%), and secondary intervention (9.5%). NLR demonstrated strong predictive efficacy (cutoff: 7.69; sensitivity: 96.4%; specificity: 65.9%; AUC = 0.83, 95% CI: 0.76–0.90). The high-NLR group exhibited significantly reduced survival compared to the low-NLR group (*P* < 0.001). Univariate Cox regression identified high NLR (HR = 30.766, 95% CI: 4.177–226.611, *P* = 0.001) and branch reconstruction (HR = 2.335, 95% CI: 1.090–5.001, *P* = 0.029) as mortality risk factors. Multivariate analysis confirmed high NLR as an independent risk factor for all-cause death (HR = 2.033, 95% CI: 1.004–3.221).

**Conclusion:**

Preoperative NLR elevation is significantly linked to decreased survival following TEVAR in AD patients with aortic dissection, and it may function as an independent predictor of all-cause mortality. This parameter may be used as a reliable prognostic marker for this specific patient group.

## 1 Introduction

Aortic dissection (AD) is a life-threatening vascular emergency characterized by exceedingly high mortality rates. Its core pathological mechanism involves medial degeneration with intimal tear formation, leading to blood extravasation into the arterial wall and subsequent false lumen development between the intimal and medial layers (Hameed et al., 2023; Juraszek et al., 2022; Sayed et al., 2021) Epidemiological studies indicate an in-hospital mortality rate of 22% for acute type A AD, contrasting with a relatively stable 14% mortality rate for acute type B AD (Mazzolai et al., 2024). Thoracic endovascular aortic repair (TEVAR) has become the first-line intervention for type B dissection due to its triple efficacy in sealing primary entry tears, promoting true lumen remodeling, and demonstrating favorable short- and long-term outcomes. Nevertheless, patients remain at significant risk of postprocedural mortality (Mazzolai et al., 2024; MacGillivray et al., 2022; Erbel et al., 2014). Consequently, identifying reliable predictors of postoperative mortality is critical for early identification of high-risk patients, enabling personalized therapeutic strategies to improve clinical outcomes.

The immune-inflammatory cascade plays a pivotal role in AD pathogenesis and progression (Cifani et al., 2015; Wu et al., 2013; He et al., 2006). The neutrophil-to-lymphocyte ratio (NLR) – an accessible, cost-effective systemic inflammatory biomarker–demonstrates proven prognostic value for cardiovascular conditions (e.g., coronary artery disease, stroke) and peripheral arterial diseases (e.g., aortic aneurysms, lower extremity arterial disease) (Monireh et al., 2025; Bradley et al., 2024; Wu et al., 2024; Evelina et al., 2024; Fengli et al., 2024; Artemio et al., 2023; Zsolt et al., 2023; Toshiya et al., 2022; Raffaela et al., 2021; Wu et al., 2025). Multiple studies have confirmed that NLR is a valuable predictor of in-hospital mortality and the occurrence of postoperative adverse events in patients with aortic dissection (Xin et al., 2025; Hanna et al., 2025; Ustaalioglu and Aydoğdu, 2024; Zhang et al., 2021; Yang et al., 2021; Zhu et al., 2021; Chung et al., 2021).

Hypertension represents the most prevalent comorbidity and an independent risk factor for AD development (Chen et al., 2025; Yang et al., 2023). Chronic hypertension can induce structural changes in the vascular wall and a chronic inflammatory environment, subjecting the aortic wall to sustained high hemodynamic stress. This pathophysiological process predisposes to intimal injury, thereby accelerating AD initiation and propagation (Mutailifu et al., 2024; Akutsu, 2019). Notably, approximately 25%–35% of AD patients present without pre-existing hypertension (Erbel et al., 2014), suggesting potential differences in inflammatory pathophysiology compared to hypertensive counterparts. However, research on the prognostic value of NLR in this specific patient subgroup (i.e., patients with AD without hypertension) remains underexplored.

Based on this background, the present study aims to investigate the potential predictive utility of preoperative NLR level for clinical outcomes in patients with non-hypertensive aortic dissection undergoing TEVAR.

## 2 Materials and methods

### 2.1 Study design and participants

This retrospective cohort study enrolled AD patients who underwent TEVAR at our medical center between December 2014 and December 2020.

Inclusion Criteria:

1. AD diagnosis confirmed by computed tomography angiography (CTA).
2. TEVAR as the primary treatment.
3. Signed informed consent approved by the Institutional Review Board (IRB).

Exclusion Criteria:

1. History of malignancy.
2. Active infection.
3. Recent use of anti-inflammatory medications (within 4 weeks).
4. Hematologic or autoimmune disorders.
5. Diagnosis of Marfan syndrome.
6. Incomplete key clinical data.

**FIGURE 1:**
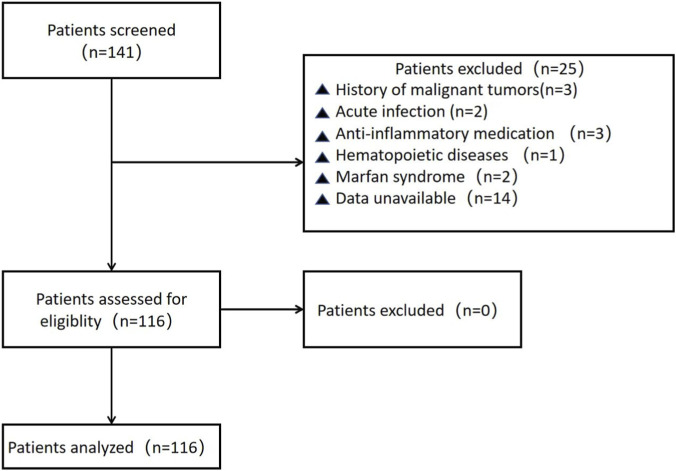
Flow chart of study.

Among 141 initially eligible patients, 25 were excluded (malignancy: n = 3; active infection: n = 2; anti-inflammatory use: n = 3; hematologic/autoimmune disorders: n = 1; Marfan syndrome: n = 2; missing data: n = 14). Consequently, 116 patients were included in the final analysis (Figure 1). The study protocol was approved by the Ethics Committee of our medical center, and written informed consent was obtained from all participants.

### 2.2 Data collection and definitions

Baseline clinical characteristics, imaging data, operative records, and outcomes were extracted from electronic medical records.

Anatomical classification: Stanford type A (ascending aorta involvement) or type B (isolated descending aorta involvement) (Mazzolai et al., 2024). Classification of timing: hyperacute (<24 h), acute (1–14 days), subacute (15–90 days), or chronic (>90 days) based on symptom-to-diagnosis interval (Mazzolai et al., 2024).

In this study, “non-hypertensive” was defined as the absence of a prior hypertension diagnosis and no regular use of antihypertensive medications before the onset of aortic dissection. Because admission blood pressure in patients with acute aortic dissection often fluctuates considerably due to pain, stress, or hemodynamic instability, a single blood pressure measurement upon admission does not reliably reflect the patient’s baseline blood pressure status. Therefore, measured admission blood pressure values were not used as the basis for classification. The hypertension history was determined according to prior diagnostic records and medication histories documented in the electronic medical records.

#### 2.2.1 Blood tests and NLR calculation

Venous blood samples were collected preoperatively using a tourniquet. Absolute neutrophil and lymphocyte counts were measured via automated hematology analyzer. The neutrophil-to-lymphocyte ratio (NLR) was calculated as: NLR = Absolute Neutrophil Count ​/Absolute Lymphocyte Count.

#### 2.2.2 Clinical variables

Assessed parameters included:

1. Demographics: Age, sex, smoking/alcohol history.
2. Comorbidities:Diabetes, renal insufficiency, ischemic heart disease, cerebrovascular disease, COPD.
3. AD-Specific Variables: Stanford classification and temporal classification.

### 2.3 Treatment and follow-up

All patients underwent TEVAR.

Primary Endpoint: all-cause mortality (perioperative [≤30 days] and long-term).

Secondary Endpoints: endoleak, secondary intervention.

Follow-up: regular outpatient visits or telephone interviews through 30 December 2024. Survival time was calculated from surgery to death or last follow-up.

### 2.4 Statistical analysis

Continuous variables underwent normality testing (*Shapiro-Wilk* test), expressed as mean ± SD (normal distribution) or median [IQR] (non-normal distribution). Categorical variables were reported as frequencies (%), compared using *χ* ^*2*^ or Fisher’s exact test (expected cell count <5). Group comparisons for continuous variables used independent *t*-test (normal distribution, equal variance) or *Mann-Whitney U* test (non-normal distribution). Receiver operating characteristic (ROC) curve analysis determined the optimal NLR cutoff (maximizing Youden’s index: sensitivity + specificity−1), AUC, and 95% CI. Patients were stratified into low/high-NLR groups based on this cutoff. Survival differences were analyzed using *Kaplan-Meier* curves with *log-rank* testing. Univariate Cox regression identified mortality-associated variables; covariates with *P* < 0.10 or clinical relevance entered multivariate Cox regression to identify independent predictors. Results were reported as hazard ratio (HR) with 95% CI. All analyses used GraphPad Prism 9.0 (La Jolla, CA) and IBM SPSS 26.0 (Armonk, NY), with two-sided *P* < 0.05 indicating statistical significance.

## 3 Results

### 3.1 Patient baseline characteristics

Of the 116 patients, 93 (80.2%) were followed up via outpatient visits and 23 (19.8%) via telephone interviews. The follow-up completion rate was 100%, with no patients lost to follow-up. The median follow-up duration was 65 months (range, 1–137 months).

This study enrolled 116 AD patients without hypertension. Baseline characteristics are detailed in Table 1. The cohort comprised 77.6% males (90/116), 52.6% aged >55 years (61/116), 30.2% with smoking history (35/116), and 13.8% with alcohol use (16/116). Comorbidities were present in 14.7% (17/116), including diabetes (3.4%, n = 4), renal insufficiency (2.6%, n = 3), ischemic heart disease (3.4%, n = 4), cerebrovascular disease (3.4%, n = 4), and COPD (1.7%, n = 2).

**TABLE 1:** Demographic data.

Parameters	N. of patients (%)
Age>55	61 (52.6%)
Female	26 (22.4%)
Smoking	35 (30.2%)
Alcohol use	16 (13.8%)
Comorbidities	17 (14.7%)
Diabetes	4 (3.4%)
Renal insufficiency	3 (2.6%)
Ischemic heart disease	4 (3.4%)
Cerebrovascular disease	4 (3.4%)
COPD	2 (1.7%)

### 3.2 Dissection characteristics, branch reconstruction, and postoperative outcomes

Stanford classification included type A (5.2%, 6/116) and type B (94.8%, 110/116). Disease stages were acute (12.1%, 14/116), subacute (30.2%, 35/116), and chronic (57.8%, 67/116). Branch artery reconstruction was performed in 47.4% (55/116), utilizing chimney technique (8.6%, 10/116), *in vitro* fenestration (17.2%, 20/116), or branched stent implantation (21.6%, 25/116). Postoperative outcomes included all-cause mortality (1.7%, 2/116), dissection recurrence (3.4%, 4/116), endoleak (6.0%, 7/116), and secondary intervention (9.5%, 11/116) (Table 2).

**TABLE 2:** Dissection characteristic, branch reconstruction, and postoperative outcomes.

Variable	N. of patients (%)
Stanford classifications	
A	6 (5.2%)
B	110 (94.8%)
Temporal classifications	
Acute	14 (12.1%)
Subacute	35 (30.2%)
Chronic	67 (57.8%)
Branch reconstruction	
Yes	55 (47.4%)
No	61 (52.6%)
Reconstruction methods	
Chimney	10 (8.6%)
*In vitro* fenestration	20 (17.2%)
Branch stent implantation	25 (21.6%)
Postoperative outcomes	
Dissection recurrence	4 (3.4%)
Endoleak	7 (6.0%)
Secondary intervention	11 (9.5%)
All-cause mortality	2 (1.7%)

### 3.3 ROC curve analysis of NLR for predicting outcomes

**FIGURE 2:**
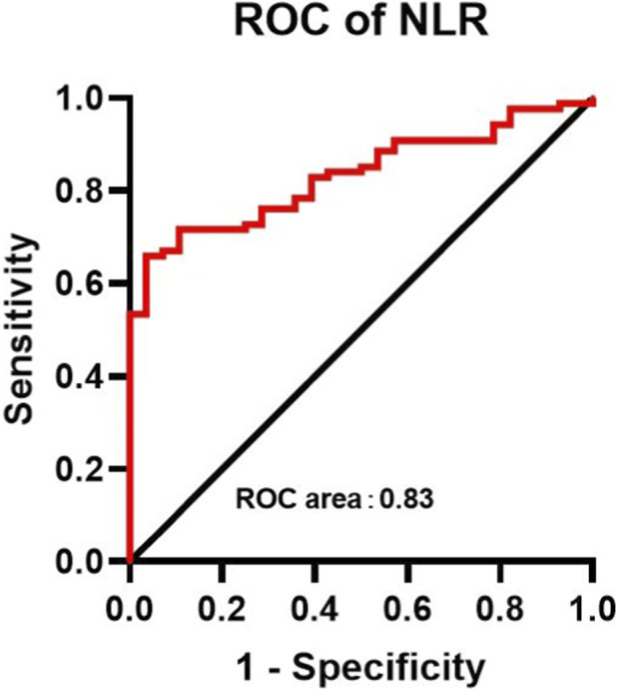
ROC curve analysis of NLR for predicting outcomes.

ROC curve analysis (Figure 2) demonstrated NLR’s predictive efficacy for all-cause mortality: optimal cutoff = 7.69, sensitivity = 96.4%, specificity = 65.9%, AUC = 0.83 (95% CI: 0.76–0.90). These results indicate strong predictive value of preoperative NLR for post-TEVAR mortality in non-hypertensive AD patients.

### 3.4 Association between NLR groups and baseline characteristics

Patients were stratified into low-NLR (n = 59) and high-NLR (n = 57) groups using the NLR cutoff (7.69). Univariate analysis (Table 3) revealed smoking history was significantly associated with high NLR (*P* = 0.035). No other baseline characteristics (age, sex, alcohol use, comorbidities, AD classification/staging) differed between groups (*P* > 0.05).

**TABLE 3:** Correlation of NLR with patient’s clinical features.

Clinical characteristics	NLR		
	Low	High	P
Age(years)	​	​	0.26
<55	31	24	​
≥55	28	33	​
Gender	​	​	0.429
Female	15	11	​
Male	44	46	​
Smoking	​	​	0.035
Absent	45	36	​
Present	12	23	​
Alcohol use	​	​	0.316
Absent	49	51	​
Present	10	6	​
Diabetes	​	​	0.326
Absent	56	56	​
Present	3	1	​
Chronic kidney disease	​	​	0.538
Absent	58	55	​
Present	1	2	​
Cerebrovascular disease	​	​	0.326
Absent	56	56	​
Present	3	1	​
Ischemic heart disease	​	​	0.326
Absent	56	56	​
Present	3	1	​
COPD	​	​	0.98
Absent	58	56	​
Present	1	1	​

### 3.5 Survival analysis

**FIGURE 3:**
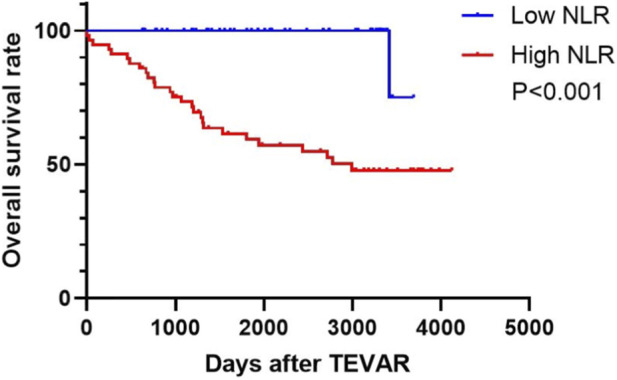
Kaplan-Meier curves with log-rank testing.

*Kaplan-Meier* curves (Figure 3) showed significantly lower survival in high-NLR versus low-NLR groups (*P* < 0.001). Univariate Cox regression identified high NLR (HR = 30.766, 95% CI: 4.177–226.611, P = 0.001) and branch reconstruction (HR = 2.335, 95% CI: 1.090–5.001, P = 0.029) as mortality risk factors. Multivariate Cox analysis confirmed preoperative high NLR as an independent risk factor for all-cause death (HR = 2.033, 95% CI: 1.004–3.221) (See Table 4).

**TABLE 4:** Univariate and multivariate analyses of characteristics with overall survival.

Variable	Univariate	*p*	Multivariate	*p*
	HR (95% CI)		HR (95% CI)	
Age (<55 years vs. ≥ 55 years)	1.815 (0.820–4.017)	0.142	​	​
Gender (male vs. female)	1.736 (0.602–5.005)	0.307	​	​
Smoking (absent vs. present)	0.589 (0.198–1.755)	0.342	​	​
Alcohol use (absent vs. present)	0.865 (0.170–4.397)	0.862	​	​
Diabetes (absent vs. present)	1.050 (0.142–7.742)	0.962	​	​
Chronic kidney disease (absent vs. present)	1.739 (0.234–12.920)	0.589	​	​
Cerebrovascular disease (absent vs. present)	0.047 (0.000–203.896)	0.474	​	​
Ischemic heart disease (absent vs. present)	0.046 (0.000–124.691)	0.446	​	​
COPD(absent vs. present)	7.022 (0.900–54.806)	0.063	​	​
Stanford classification	0.575 (0.078–4.242)	0.587	​	​
Intervention phase	1.185 (0.690–2.034)	0.539	​	​
Branch reconstruction	2.335 (1.090–5.001)	0.029	​	​
NLR(low vs. high)	30.766 (4.177–226.611)	0.001	2.033 (1.004–3.221)	0.001

## 4 Discussion

This retrospective study demonstrates that preoperative NLR independently predicts all-cause mortality in non-hypertensive AD patients undergoing TEVAR, as evidenced by multivariate Cox regression analysis. The ROC curve further supports NLR’s utility as a prognostic biomarker for postoperative mortality risk in this specific cohort.

The immune-inflammatory response plays a significant role in AD pathogenesis (Cifani et al., 2015; He et al., 2006). Histologically, AD is characterized by medial degeneration with extensive inflammatory cell infiltration (Wu et al., 2013; He et al., 2006), where heightened inflammation correlates strongly with adverse outcomes (Kato et al., 2010). Neutrophil and lymphocyte dynamics reflect disease progression: elevated neutrophil counts independently predict in-hospital mortality in acute type A AD (Feng et al., 2023), while immature neutrophils associate with adverse events following TEVAR in patients with uncomplicated type B AD (Abu et al., 2023). Conversely, patients with acute type A AD exhibit a reduction in peripheral blood T lymphocytes (del Porto et al., 2010), and lymphopenia correlates with poor outcomes in critical conditions including AD (Xin et al., 2025; Yang et al., 2021; Heffernan et al., 2012). However, single leukocyte parameters (such as neutrophil or lymphocyte counts) are susceptible to interference from hydration status and sample processing, which limits their clinical utility (Yang et al., 2021).

As a systemic inflammatory biomarker, NLR integrates innate (neutrophils) and adaptive (lymphocytes) immune responses, providing a stable and clinically practical assessment. Its ratio-based format demonstrates superior sensitivity to single parameter while remaining cost-effective and readily accessible (Xin et al., 2025; Yang et al., 2021). Multiple studies have demonstrated the prognostic value of the NLR in AD. A systematic review has confirmed that elevated NLR is associated with increased in-hospital mortality in aortic diseases, including AD and aortic aneurysms, and significantly raises the risk of postoperative mortality in patients undergoing surgical repair for aortic disease (Xu et al., 2020). Another systematic review has highlighted that NLR has predictive utility for in-hospital mortality following surgery in patients with type A AD (Chung et al., 2021). Clinical studies have further established that elevated preoperative NLR is significantly associated with increased occurrence of early adverse events and decreased survival rates following surgical repair of aortic dissection (Xin et al., 2025; Hanna et al., 2025; Ustaalioglu and Aydoğdu, 2024; Bedel and Selvi, 2019). Moreover, in the emergency setting, NLR can effectively differentiate aortic dissection (AD) from other acute chest pain conditions. As an independent predictor of in-hospital mortality for patients with aortic dissection, an elevated NLR value indicates an increased risk of in-hospital death (Zhang et al., 2021). Through propensity score matching analysis, Yang et al. found that elevated NLR is an independent predictor of in-hospital and follow-up mortality following TEVAR in patients with type B aortic dissection (Yang et al., 2021).

The NLR cutoff value derived from this study is largely consistent with that reported in the broader vascular surgery population. A meta-analysis of aortic diseases reported that NLR cutoff values across studies ranged from 3.5 to 9.7 (36), and the cutoff of 7.69 identified in the present study falls within this range. Recent systematic reviews have further supported the prognostic value of NLR: it predicts all-cause mortality in patients with abdominal aortic aneurysms undergoing open surgical repair (OSR) or endovascular aneurysm repair (EVAR) (Bradley et al., 2025), and the latest meta-analysis encompassing 1,792 AD patients confirmed that elevated NLR is associated with an increased risk of mortality (Li et al., 2025). These findings indicate that NLR demonstrates considerable consistency across different aortic diseases and surgical modalities, thereby supporting its utility in the present study population.

Hypertension, a major risk factor and common comorbidity for AD, contributes to its pathogenesis through multiple pathways: chronic hypertension can lead to degenerative changes in the aortic media, promote atherosclerotic lesions, and increase mechanical shear stress on the aortic wall, thereby creating a pathological basis for the occurrence of AD and directly inducing the formation of intimal tears. Hypertension is a principal comorbidity of aortic dissection and one of the most significant risk factors (Sayed et al., 2021; Akutsu, 2019; Suzuki et al., 2022; Dong et al., 2019). It is noteworthy that approximately 25%–35% of patients with AD do not have a history of hypertension before the onset of the disease (Erbel et al., 2014), yet research on inflammatory markers in this subgroup is relatively limited. This study fills this gap by focusing on patients with AD who do not have hypertension and investigating the value of NLR in predicting the outcomes of TEVAR in this population.

## 5 Limitations

This study has several limitations. First, the optimal NLR cutoff (7.69) was determined by ROC curve analysis using Youden’s index. When the number of events is limited, such a cutoff may be unstable and subject to optimism; furthermore, dichotomization of a continuous variable reduces statistical power and may obscure dose-response relationships. Therefore, this threshold should be regarded as an exploratory finding in this cohort. Second, constrained by the limited number of events, the events-per-variable ratio fell below the widely accepted minimum threshold of 10. Consequently, the multivariate Cox model included only the primary variable (NLR) and did not incorporate covariates such as branch reconstruction. The model may therefore be susceptible to overfitting, and the reported hazard ratios (HRs) and 95% confidence intervals (CIs) should be interpreted with caution. Third, owing to the single-center retrospective design, this study was unable to capture surgical parameters beyond standard TEVAR procedures and branch reconstruction techniques, including stent-graft brand/type, left subclavian artery management, proximal landing zone, and extent of aortic coverage. These factors may influence prognosis and constitute potential confounders in the association between NLR and all-cause mortality. These limitations warrant validation through prospective studies with larger sample sizes and more comprehensive data.

## 6 Conclusion

This study confirmed that elevated preoperative NLR is significantly associated with decreased postoperative survival in patients with AD without hypertension undergoing TEVAR. Multivariate analysis indicated that high NLR is an independent risk factor for all-cause mortality following TEVAR in these specific patients. Thus, preoperative NLR may serve as an effective prognostic indicator for these patients, providing a basis for clinical risk stratification and individualized treatment decisions.

## Data Availability

The datasets used and/or analyzed during the current study are available from the corresponding author on reasonable request.
